# The Impact of Temperature and Pressure on the Structural Stability of Solvated Solid-State Conformations of *Bombyx mori* Silk Fibroins: Insights from Molecular Dynamics Simulations

**DOI:** 10.3390/ma17235686

**Published:** 2024-11-21

**Authors:** Ezekiel Edward Nettey-Oppong, Riaz Muhammad, Ahmed Ali, Hyun-Woo Jeong, Young-Seek Seok, Seong-Wan Kim, Seung Ho Choi

**Affiliations:** 1 Department of Biomedical Engineering, Yonsei University, Wonju 26493, Republic of Korea; ezekieledward@yonsei.ac.kr (E.E.N.-O.); riaz@yonsei.ac.kr (R.M.); alee@yonsei.ac.kr (A.A.); 2 Department of Electrical Engineering, Sukkur IBA University, Sukkur 65200, Pakistan; 3 Department of Biomedical Engineering, Eulji University, Seongnam 13135, Republic of Korea; hwjeong@eulji.ac.kr; 4 Gangwon-do Agricultural Product Registered Seed Station, Chuncheon 24410, Republic of Korea; air5738@korea.kr; 5 Department of Agricultural Biology, National Institute of Agricultural Sciences, Rural Development Administration, Wanju 55365, Republic of Korea; 6 Department of Integrative Medicine, Major in Digital Healthcare, Yonsei University College of Medicine, Seoul 06229, Republic of Korea

**Keywords:** *Bombyx mori*, molecular dynamics, structural stability, thermal agitation

## Abstract

*Bombyx mori* silk fibroin is a promising biopolymer with notable mechanical strength, biocompatibility, and potential for diverse biomedical applications, such as tissue engineering scaffolds, and drug delivery. These properties are intrinsically linked to the structural characteristics of silk fibroin, making it essential to understand its molecular stability under varying environmental conditions. This study employed molecular dynamics simulations to examine the structural stability of silk I and silk II conformations of silk fibroin under changes in temperature (298 K to 378 K) and pressure (0.1 MPa to 700 MPa). Key parameters, including Root Mean Square Deviation (RMSD), Root Mean Square Fluctuation (RMSF), and Radius of Gyration (R_g_) were analyzed, along with non-bonded interactions such as van der Waals and electrostatic potential energy. Our findings demonstrate that both temperature and pressure exert a destabilizing effect on silk fibroin, with silk I exhibiting a higher susceptibility to destabilization compared to silk II. Additionally, pressure elevated the van der Waals energy in silk I, while temperature led to a reduction. In contrast, electrostatic potential energy remained unaffected by these environmental conditions, highlighting stable long-range interactions throughout the study. Silk II’s tightly packed β-sheet structure offers greater resilience to environmental changes, while the more flexible α-helices in silk I make it more susceptible to structural perturbations. These findings provide valuable insights into the atomic-level behavior of silk fibroin, contributing to a deeper understanding of its potential for applications in environments where mechanical or thermal stress is a factor. The study underscores the importance of computational approaches in exploring protein stability and supports the continued development of silk fibroin for biomedical and engineering applications.

## 1. Introduction

Silk fibroin, a biocompatible and bioresorbable protein derived from Bombyx mori silkworms, has garnered significant interest as a versatile biomaterial for diverse biomedical applications, including drug delivery [1], tissue-engineered grafts, and scaffolds [2,3,4]. Its unique hierarchical structure, spanning from the molecular to the fiber scale, underpins its exceptional biological, chemical, mechanical, and physical properties [5]. The stability and performance of silk fibroin in biological environments are closely linked to its structural characteristics. These properties arise from the specific sequence of amino acid residues, which define its secondary and tertiary polypeptide structures, including the distinctive solid-state conformations known as silk I and silk II [6]. Understanding how these structural configurations respond to external conditions is essential to optimizing silk fibroin’s stability and expanding its applicability in environments that demand thermal and mechanical resilience.

**Figure 1:**
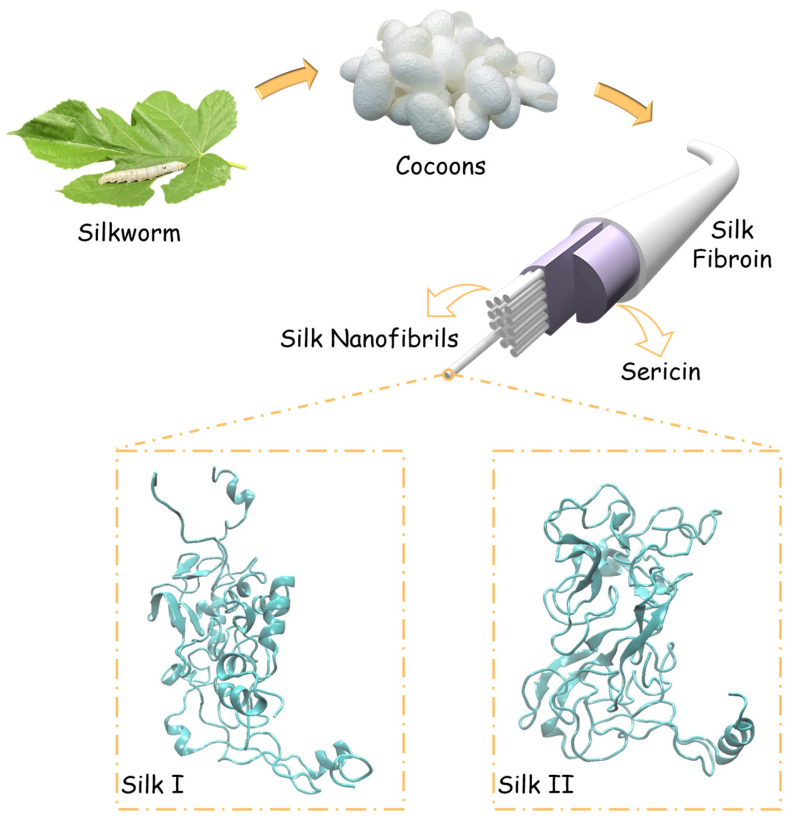
Schematic representation of *Bombyx mori* silk structure. During the pupa stage of their metamorphosis into moths, silkworms spin silk fibers to construct protective cocoons. Each silk fiber is composed of two core fibroin filaments encased by sericin (depicted in purple), an adhesive glycoprotein that facilitates fiber cohesion. At the molecular level, each fibroin filament consists of numerous assemblies of nanofibrils, which can adopt either silk I or silk II structural conformations. These structural forms are determined by the specific arrangement of secondary protein structures within the fibroin. The silk I structure predominantly features type II β-turns and α-helices, while the silk II structure is mainly characterized by β-turns and β-sheets. Both of these secondary structures contribute to the fiber’s mechanical properties and functional versatility across various applications.

Silk fibroin is a naturally occurring semicrystalline biopolymer, synthesized under mild aqueous conditions in the silk glands of *Bombyx mori* silkworms. Silk fibroin fibers produced by *Bombyx mori* are composed of core fibroin filaments, which are encased by sericin [7]. As shown in Figure 1, each fibroin filament consists of numerous silk nanofibrils. The fibroin itself is a copolymer, consisting of light and heavy chains connected via disulfide bonds, with molecular weights of approximately 26 kDa and 391 kDa, respectively [7,8,9]. Silk fibroin adopts two distinct conformations in the solid state: silk I and silk II. These modeled conformations are depicted in Figure 1. On the nanoscale, fibroin chains aggregate to form an intricate network consisting of covalently bonded crystalline and amorphous regions. The ordered phases of this structure are dispersed within a matrix of amorphous chains.

**Figure 2:**
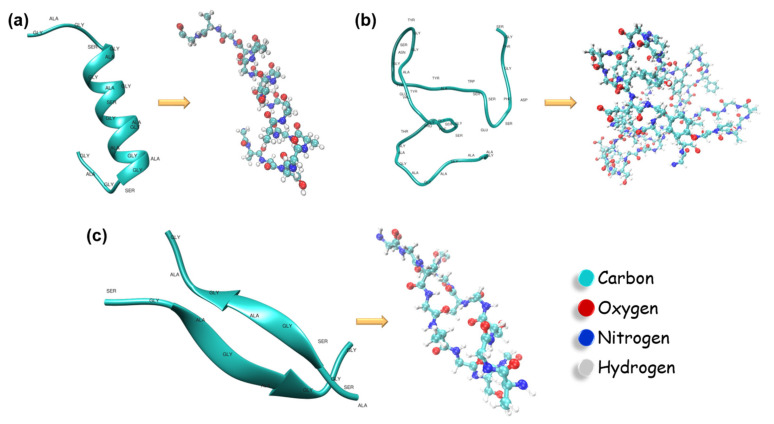
Schematic of the secondary structures of *Bombyx mori* silk fibroin. The silk fibroin protein comprises several distinct secondary structures that play a critical role in determining the material’s mechanical strength and biological properties. These secondary structures include the following: (**a**) α-helix, a right-handed coiled structure that contributes to flexibility; (**b**) β-sheet, an extended conformation that forms the crystalline regions, providing mechanical robustness; and (**c**) random coils, which are unstructured regions that contribute to the amorphous domains of the protein. The schematic also illustrates the specific amino acid residues associated with each of these secondary structures, alongside the corresponding all-atom models, providing a molecular-level perspective on silk fibroin’s hierarchical organization.

The secondary structure of silk fibroin consists of several elements—β-sheets, β-turns, helices, and random coils—depending on the species producing the silk [10]. These secondary structures can broadly be classified into crystalline and amorphous regions. The fibroin polymer chains exhibit a repetitive amino acid sequence, Gly-Ala-Gly-Ala-Gly-Ser, which predominantly forms β-sheet structures [7]. These β-sheets contribute significantly to the crystallinity of the silk and are responsible for its remarkable mechanical strength and optical properties [7,11]. In contrast, the amorphous regions are composed of non-repetitive amino acid sequences that act as linker domains between the crystalline segments [12]. Figure 2 illustrates the primary/secondary protein structures present in *Bombyx mori* silk fibroin, along with their corresponding amino acid sequences. Nuclear Magnetic Resonance (NMR) studies of silk fibroin’s solid-state structure have revealed that silk I is characterized by type II β-turns and α-helices, while silk II is primarily composed of distorted β-turns and β-sheets [10]. Both experimental and computational research has contributed to a comprehensive understanding of silk fibroin’s structural characteristics, including its amino acid sequence, chain alignment, crystallite size, and structural wrapping [6,10,11,13,14,15,16].

With rapid technological advancements, computational methods have evolved into powerful tools for elucidating the underlying mechanisms of biomolecules [17,18,19]. Atomistic simulations have proven particularly valuable in studying the structure and properties of silk fibroin at the nanoscale, overcoming many challenges associated with experimental approaches [5,20]. Among these, Molecular Dynamics (MD) simulations have been extensively employed to explore the structural and mechanical properties of silk fibroin proteins in detail [21,22,23,24,25]. Early MD models of silk fibroin structures typically simulated the various secondary structures of the protein in isolation [26]. However, some of these models were limited by a restricted amino acid composition and failed to capture critical structural features, such as β-sheets containing fewer than 100 amino acids [5,27,28]. Other models focused exclusively on the β-sheet secondary structure, primarily to investigate the mechanical properties of silk fibroin [5,27,29].

**Figure 3:**
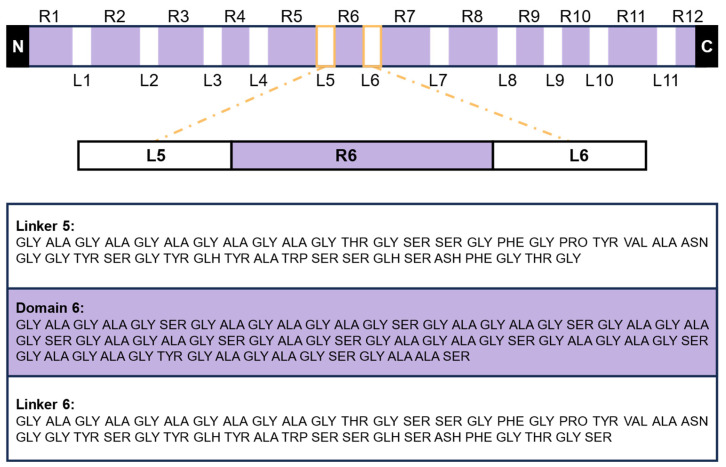
Schematic representation of the primary structure of *Bombyx mori* silk fibroin. The heavy chain of silk fibroin consists of alternating crystalline (R1 to R12) and amorphous phases (L1 to L11), along with N terminus (N) and C terminus (C), each having distinct structural and functional roles. The crystalline regions, indicated by the R domains, are primarily composed of β-sheet structures that impart rigidity and strength to the fiber. In contrast, the amorphous regions, denoted as L domains, serve as flexible linkers that provide elasticity and contribute to the material’s overall mechanical performance. This schematic highlights the representative molecular structure used for simulations, where domain R6 (crystalline) is flanked by domains L5 and L6 (amorphous), providing a balanced model for studying both phases of the fibroin.

Recently, Wongpinyochit et al. [24] developed a comprehensive and representative model of silk fibroin, which has been used to study the drug-binding and release mechanisms within silk structures. This model consists of 300 amino acids and incorporates both crystalline and amorphous regions. Structural sampling was achieved using well-tempered metadynamics, offering a robust representation of the entire silk fibroin structure. The model’s accuracy is supported by its strong correlation with structures obtained from Nuclear Magnetic Resonance (NMR) measurements [24]. The protein sequence utilized in this model is accessible from the Uniprot database under the FASTA file ID P05790. The arrangement of amino acid residues within the heavy chain of silk, along with the representative sequence domains of the modeled silk fibroin structure, is illustrated in Figure 3. Specifically, the subdomains modeled correspond to position ranges 2281–2323 for L5, 2536–2599 for R6.5, and 2600–2642 for L6.

Given the extensive use of silk fibroin in a wide range of applications, it is crucial to understand its structural responses at the atomic level, particularly in relation to external conditions. Protein structures, including silk fibroin, are influenced by variables such as temperature, pressure, and chemical environment. While some proteins exhibit denaturation or unfolding at elevated temperatures, others possess high thermal stability [30,31,32]. The response of silk fibroin to temperature variations is similarly diverse, with significant differences in structural behavior under low- versus high-temperature conditions. Pressure, too, plays a pivotal role in protein stability. For instance, Jacchetti et al. [33] conducted MD simulations to examine the structural dynamics of green fluorescent protein (GFP) mutants under various pressures. Their study revealed that the global folding of the protein becomes destabilized beyond a specific pressure threshold, while stability increased at pressures below this limit.

Studying the potential conformational and stability changes of proteins under varying temperature and pressure conditions is essential for advancing our understanding of protein structure, dynamics, and functionality. Such insights are not only fundamental from a biochemical perspective but are also highly relevant to practical applications. Protein structures are inherently sensitive to environmental conditions, and even minor perturbations in temperature and pressure can lead to significant conformational changes. These changes can, in turn, affect a protein’s mechanical properties, stability, and overall biological function. Understanding these dynamics is critical for fields such as drug design, biotechnology, and materials science.

Silk fibroin presents a unique case due to its broad application across biomedical and industrial domains. The fabrication processes for silk fibroin-based materials, whether for drug delivery systems [1], tissue engineering scaffolds, or biosensors [2,3,4], often involve exposure to extreme variations in temperature and pressure [34]. These factors can influence the structural integrity of silk fibroin, affecting its performance in final applications. However, despite its widespread use, a comprehensive understanding of how temperature and pressure impact the molecular structure of silk fibroin, especially its two dominant polymorphic forms, silk I and silk II, remains incomplete. A detailed investigation into these structural changes is therefore vital to optimizing silk fibroin for specific applications and improving the robustness of silk-based materials under different environmental conditions.

In this study, we employed Molecular Dynamics (MD) simulations to thoroughly investigate the structural stability of Bombyx mori silk fibroin in a practical aqueous environment. Specifically, we examined the effects of temperature and pressure on the stability of the solvated silk fibroin structures to understand the influence of external stressors that closely resemble those in silk processing and biomedical applications. Specifically, we examined the effects of temperature and pressure on the stability of solvated silk fibroin structures to understand the influence of external stressors that closely resemble those in silk processing and biomedical applications. By analyzing the structural stability and conformational dynamics of both silk I and silk II under these conditions, we aim to provide valuable insights into silk fibroin’s atomic-level behavior in solution, which differs from purely solid-state interactions. Additionally, we explored non-bonded interactions, such as electrostatic potential energy and van der Waals forces, to better understand how intermolecular forces contribute to maintaining silk fibroin’s stability in solution. This multifaceted approach not only highlights the resilience of silk fibroin under thermal and pressure fluctuations in an aqueous environment but also offers guidance for the design and fabrication of silk-based materials with enhanced performance for diverse applications.

## 2. Computational Method

### 2.1. Molecular Modeling

Molecular dynamics (MD) simulations were conducted using the Large-scale Atomic/Molecular Massively Parallel Simulator (LAMMPS) package [35] (https://www.lammps.org, access date: 31 August 2021). Before initiating the MD simulations, hydration was modeled with the CHARMM-GUI interface [36,37] (https://charmm-gui.org/, access date: 3 October 2022), employing the TIP3P water model. The silk fibroin structures were immersed in a cubic simulation box containing 35,602 water molecules for silk I and 21,159 water molecules for silk II, ensuring an edge distance of 10 Å between the silk structures and the box boundaries. The final dimensions of the simulation cells were 10.5 × 10.5 × 10.5 nm^3^ for silk I and 8.9 × 8.9 × 8.9 nm^3^ for silk II, providing adequate space for molecular interactions and minimizing boundary effects.

### 2.2. Simulation Details

LAMMPS was employed to carry out energy minimization, equilibration, and dynamics simulations of the silk fibroin structures under varying temperature and pressure conditions. The CHARMM 36 m force field was utilized to model interatomic interactions [38], ensuring the accuracy of atomic interactions in the silk fibroin system. The CHARMM force field is extensively validated in MD simulations and has been proven to accurately represent the structural dynamics of biomolecules [39,40,41,42]. To handle the pairwise atomic interactions, the most recent version of the CHARMM force field was applied, incorporating force-switching and force-shifting functions for Lennard-Jones (LJ) and Coulombic interactions, rather than relying on traditional energy-switching techniques [43].

For long-range interactions, the particle–particle–particle–mesh (PPPM) solver [44] was utilized to compute Coulombic forces. The PPPM solver applies 3D fast Fourier transforms (FFTs) to solve Poisson’s equation over a 3D mesh, mapping atomic charges onto the mesh and interpolating electric fields back to the atoms. The use of FFTs allows the PPPM solver to scale as Nlog(N), where N is the total number of atoms, offering a computationally efficient alternative to the conventional Ewald summation method, which scales as N^3/2^ [45]. To simulate an infinite system, periodic boundary conditions (PBCs) were applied in all directions. Energy minimization was conducted using the conjugate gradient (CG) method, specifically the Polak–Ribiere variant. The CG algorithm iteratively adjusts atom coordinates by coupling the force gradient with information from the previous iteration, determining new search directions that are conjugate (perpendicular) to the previous direction. This method allows for the configuration to iteratively approach a local potential energy minimum, resulting in a stable molecular configuration for further simulations.

**Figure 4:**
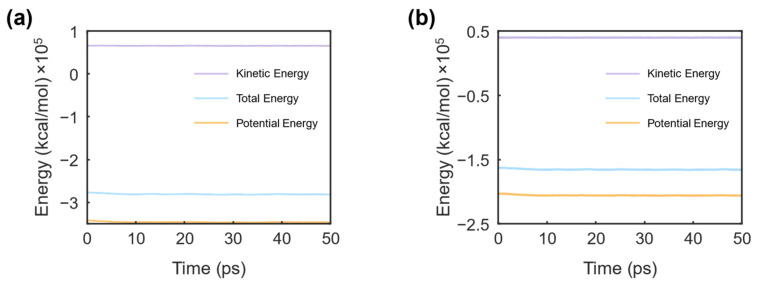
Molecular dynamics equilibration simulation of silk fibroin protein structures. This figure illustrates the evolution of both potential energy and kinetic energy over time during the equilibration phase for hydrated silk fibroin systems. Equilibration was performed under the NPT ensemble for the silk I (**a**) and silk II (**b**) structures. The gradual decrease and stabilization of the total potential energy throughout the simulation indicates that the system achieves a stable configuration as the atoms settle into their equilibrium positions. The kinetic energy, in contrast, remains relatively constant, reflecting consistent thermal motion within the system. This energy evolution demonstrates the successful stabilization of silk fibroin structures under the specified conditions, preparing them for further simulation analyses.

**Figure 5:**
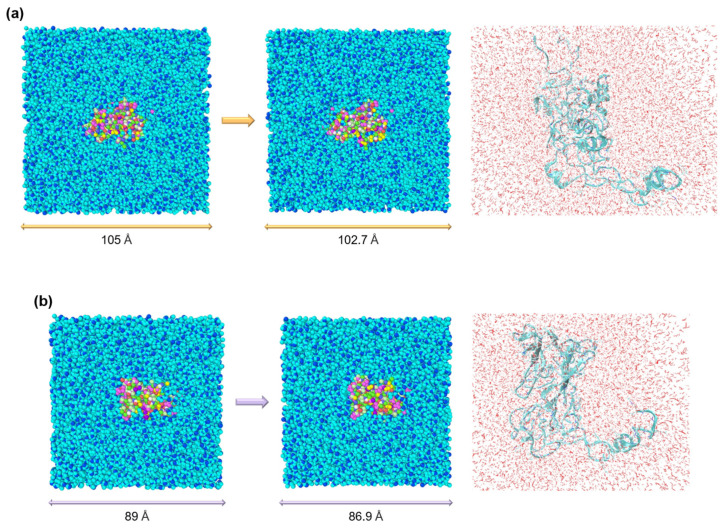
Volume changes of the simulation cells during equilibration of silk fibroin structures. The figure presents the observed reduction in the volume of the simulation cells during equilibration. For the silk I structure (**a**), the cubic cell size decreased from an initial length of 105 Å to 102.7 Å, while for the silk II structure (**b**), the cell size reduced from 89 Å to 86.9 Å. This volume contraction is indicative of the system reaching equilibrium, as the protein and water molecules reorganize into a more compact and energetically favorable configuration. The simulations ensured complete hydration of the protein by maintaining a 10 Å buffer between the protein structures and the cell boundaries. A visual snapshot on the far right provides a cross-sectional view of the hydrated silk I and silk II proteins, illustrating the distribution of water molecules around the protein structures and confirming that the proteins are fully solvated within the simulation cells.

Following energy minimization, each system underwent equilibration in both the NVT and NPT ensembles. Newton’s equations of motion were integrated using the velocity-Verlet scheme with a timestep of 0.1 fs. An equilibration at 298 K was performed in the NVT ensemble, while the NPT ensemble maintained a pressure of 1 atm. During equilibration simulations, Nose−Hoover style non-Hamiltonian equations of motion were integrated to sample velocities and positions from the canonical (NVT) and isothermal–isobaric (NPT) ensembles. Bonds involving hydrogen atoms were constrained using the SHAKE algorithm [46], ensuring accurate hydrogen-bond modeling. The Nose–Hoover thermostat and barostat were used to regulate temperature and pressure, respectively. Figure 4 shows the energy evolution during the NPT equilibration of the simulated systems, where a gradual reduction in potential energy was observed, indicating stabilization of the silk fibroin structures. In contrast, the kinetic energy remained constant throughout the simulation timesteps. Correspondingly, the simulation cell experienced a reduction in volume, as illustrated in Figure 5. For silk I, the cubic cell size reduced from 105 Å to 102.7 Å, and for silk II, from 89 Å to 86.9 Å, signifying the structural stabilization of both silk I and silk II.

### 2.3. Variation of Temperature and Pressure

Subsequent simulations explored the effects of varying temperature and pressure on the silk fibroin structures under NVT and NPT ensembles. The temperature was varied between 298 K and 378 K, while pressure ranged from 0.1 MPa to 700 MPa. Additionally, the non-bonded interactions, including van der Waals energy and electrostatic potential energy, were computed to investigate their influence on silk fibroin stability under these conditions. Structural visualization and stability analyses of the silk fibroin proteins under different temperature and pressure regimes were conducted using Visual Molecular Dynamics (VMD) [47], UCSF Chimera [48], and OVITO [49]. These tools enabled detailed insights into the conformational dynamics and structural integrity of silk fibroin in response to external stimuli, providing a comprehensive understanding of its behavior at the molecular level.

### 2.4. Structural and Conformational Analysis

To thoroughly analyze the dynamic behavior of the silk fibroin protein structures under varying temperature and pressure conditions, several key structural parameters were quantified, including Root Mean Square Deviation (RMSD), Root Mean Square Fluctuation (RMSF), and Radius of Gyration (R_g_). These metrics are widely used to assess the stability and conformational flexibility of proteins under different simulation conditions [50,51,52,53,54].

Root Mean Square Deviation (RMSD) is a critical parameter for evaluating the overall conformational changes of a protein structure over time. It measures the average deviation between the initial and final atomic coordinates, providing insights into how the structure evolves during the simulation. Notably, the Kabsch algorithm was applied during RMSD calculations to achieve optimal alignment [55,56]. This alignment correction minimizes the impact of pure rotational and translational movements, allowing the RMSD values to accurately capture the structural deviations and true conformational changes in the protein rather than artifacts caused by pure rotation. RMSD is particularly useful for identifying significant structural rearrangements or shifts from the initial conformation. The RMSD is calculated as the following:

(1) RMSD=1n∑i=1nrfi−rii212

where *n* is the number of protein atoms, $r_{i}$ and $r_{f}$ are the coordinates of an atom *i* of the initial structure and final structure, respectively. By analyzing the RMSD, we can infer the degree of structural stability or flexibility of the silk fibroin under different conditions, with higher RMSD values typically indicating greater deviation and flexibility in the protein structure.

Root Mean Square Fluctuation (RMSF), on the other hand, is a metric used to assess the flexibility of individual atoms, particularly the alpha carbon (C^α^) atoms, within the protein structure. It measures the degree of fluctuation of atomic positions relative to the reference structure (usually the initial configuration) over the course of the simulation. RMSF is useful for identifying flexible regions, such as loops or unstructured domains, that exhibit higher atomic mobility. The RMSF is calculated as the following:

(2) RMSFi=1T∑tj=1Tritj−riref212

where *T* is the total simulation time, and *r^ref^* is the average coordinate of the C^α^ atom for the entire simulation time. RMSF helps in pinpointing the specific regions of the protein that experience significant fluctuations, which may correspond to functional or structurally important domains.

The Radius of Gyration (R_g_) is another essential parameter that provides insights into the compactness of the protein structure. It measures the distribution of atoms relative to the center of mass of the protein and reflects how the structure expands or contracts under varying conditions. A decrease in R_g_ may suggest a more compact and stable structure, while an increase indicates expansion or unfolding. The Radius of Gyration is calculated as the following:

(3) Rg=1n∑i=1nri−rc212

where *n* is the number of protein atoms, $r_{i}$ is the coordinates of atom *i*, and $r_{c}$ is the center of mass of the structure. Together, RMSD, RMSF, and R_g_ provide a comprehensive view of the structural stability, flexibility, and compactness of the protein under different simulation conditions. These metrics are instrumental in evaluating how external factors such as temperature and pressure influence the conformational dynamics of silk fibroin, enabling a deeper understanding of its molecular behavior in various environments.

## 3. Results and Discussion

The structural stability of silk fibroin, specifically solvated structures of silk I and silk II conformations, under varying temperature and pressure conditions, was characterized by evaluating these key structural parameters: Root Mean Square Deviation (RMSD), Root Mean Square Fluctuation (RMSF), and Radius of Gyration (R_g_). These metrics provide critical insights into the conformational dynamics and stability of the macromolecule under different environmental stimuli. Additionally, the Instability Index (II) of the silk fibroin protein structure was computed using the Expasy ProtParam tool [57] (https://web.expasy.org/protparam/, access date: 13 November 2022), which assesses the chemical and physical parameters of proteins to predict their stability. The instability index for silk fibroin was found to be 33.26, approximately 7 units below the instability threshold of 40, signifying that the protein structure is inherently stable under normal conditions [58,59]. A protein with an instability index below 40 is considered stable, while values above this threshold indicate potential structural instability. Thus, the initial configurations of both silk I and silk II were stable before subjecting them to Molecular Dynamics (MD) simulations under varying temperature and pressure conditions.

Silk I and silk II conformations can be effectively distinguished by their dominant secondary structures: silk I predominantly consists of α-helices, while silk II is characterized by a higher content of β-sheets (see Figure 1). The models employed in this study for silk I and silk II are based on well-established structures that have been validated through experimental techniques, including Nuclear Magnetic Resonance (NMR) measurements [24]. This prior validation strengthens the accuracy of our simulations, allowing us to reliably interpret the stability differences between silk I and silk II under varying environmental conditions. Variations in external factors such as temperature and pressure are known to significantly influence protein stability, potentially enhancing, diminishing, or preserving structural integrity depending on the severity of the conditions. Moreover, non-bonded interactions within the silk fibroin, such as the van der Waals and electrostatic forces, also play an essential role in maintaining the structural configuration. These interactions were analyzed by examining changes in the system’s energy profile during simulation. The dynamic behavior of the silk fibroin structures is discussed below.

### 3.1. RMSD Analysis of Silk Structures

Root Mean Square Deviation (RMSD) was used to assess the overall conformational stability of the silk fibroin structures (silk I and silk II) under varying temperature and pressure conditions. RMSD values reflect the perturbations experienced by the C^α^ backbone atoms of the protein, providing a quantitative measure of how much the structure deviates from its initial conformation.

#### 3.1.1. Effect of Pressure on C^α^ Atoms Deviations

**Figure 6:**
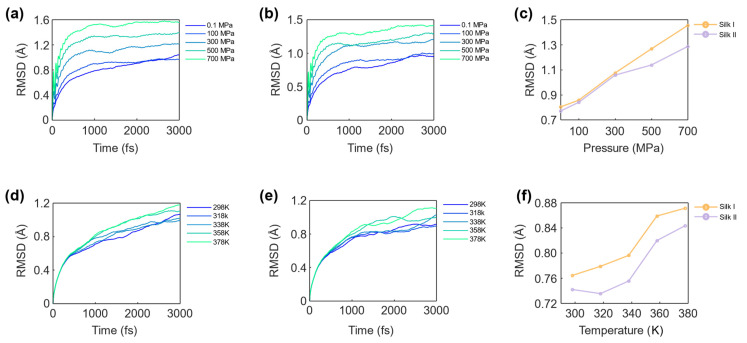
Root Mean Square Deviation (RMSD) of silk I and silk II structures under different pressure and temperature conditions. (**a**) RMSD values for the backbone atoms of silk I across a pressure range of 0.1 MPa to 700 MPa, showing how the structural deviation increases with pressure. (**b**) RMSD values for the backbone atoms of silk II under the same pressure range. Both structures exhibit increasing deviation with pressure. (**c**) The average RMSD values for silk I and silk II as a function of pressure. Silk I has a minimum deviation of 0.804 Å at 0.1 MPa, increasing to a maximum of 1.454 Å at 700 MPa. Similarly, silk II exhibits a minimum deviation of 0.772 Å at 0.1 MPa and a maximum of 1.285 Å at 700 MPa. These results demonstrate that pressure induces structural instability, with silk I experiencing a more pronounced deviation than silk II. (**d**) RMSD of silk I backbone atoms across a temperature range of 298 K to 378 K, indicating how thermal agitation affects structural deviation. (**e**) RMSD values for silk II at the same temperature range, illustrating the temperature-dependent structural perturbations. (**f**) The average RMSD values for silk I and silk II as a function of temperature. The temperature increase caused a rise in RMSD for both structures, with silk I showing a deviation from 0.764 Å at 298 K to 0.871 Å at 378 K, and silk II deviating from 0.742 Å to 0.843 Å over the same temperature range. This increase indicates that thermal agitation leads to greater atomic movement and structural deviations, particularly for silk I.

The impact of pressure on the structural deviations of the silk fibroin was investigated, with RMSD values plotted over time for pressures ranging from 0.1 MPa to 700 MPa. As shown in Figure 6a for the silk I structure and Figure 6b for the silk II structure, RMSD values steadily increase with rising pressure. A comparative analysis is provided in Figure 6c, which illustrates the average RMSD values for both structures at the different pressures simulated. At 0.1 MPa, the RMSD values for silk I and silk II were 0.804 Å and 0.772 Å, respectively, indicating minimal structural perturbations under low pressure. However, at 700 MPa, these values rose to 1.454 Å and 1.285 Å, demonstrating a significant increase in atomic deviations. This increase in RMSD correlates with a decline in structural stability as pressure rises, implying that the silk fibroin structures are destabilized by high-pressure conditions. The greater deviation observed in the silk I structure, compared to silk II, suggests that silk I is more susceptible to pressure-induced structural changes. The relative resilience of silk II may be attributed to its β-sheet-dominated structure, which provides enhanced mechanical stability compared to the more flexible α-helix and β-turn motifs found in silk I. Thus, the findings indicate that silk fibroin’s mechanical properties, particularly under high-pressure environments, are highly dependent on its conformation, with silk II offering greater resistance to such perturbations.

Pressure-induced perturbations in silk fibroin likely disrupt the non-bonded interactions between atoms, particularly the van der Waals forces and electrostatic interactions that help maintain structural stability under normal conditions. Silk fibroin’s β-sheet structure, particularly in silk II, is reinforced by tightly packed, repetitive hydrophobic regions within its heavy chains. These regions form stable, anti-parallel β-sheet crystalline structures supported by van der Waals forces, hydrogen bonding, and hydrophobic interactions, which collectively strengthen the β-sheet arrangement and confer mechanical durability [60]. Van der Waals forces play a central role in compacting these hydrophobic domains, contributing to a crystalline conformation that is essential for silk fibroin’s mechanical strength. Van der Waals forces, though relatively weak, arise from induced polarization effects between atoms and molecules in close proximity, creating attraction forces that significantly impact protein structure and stability [61,62]. These interactions are often represented by the Lennard-Jones potential, which models the balance between short-range repulsion and long-range attraction critical to maintaining structural coherence. When van der Waals forces are disrupted, this can lead to suboptimal packing within the protein structure, which may subsequently weaken or improve the overall protein stability [61,63].

In silk fibroin, the presence of polar carboxyl and amino groups within amino acid residues introduces electrostatic interactions that contribute further to the stability of the protein. These groups enable dipole-dipole interactions and, in certain cases, can participate in proton transfer [64], creating localized electrostatic interactions that stabilize the structure by enhancing cohesion within the molecular framework. These electrostatic interactions can also be disrupted under high pressure if the structural conformation changes enough to alter the spatial arrangement of charged residues leading to a pressure-induced ion-pair dissociation [61]. These mechanisms have been observed in various protein structures where high-pressure conditions disrupt van der Waals forces and electrostatic interactions, impacting the protein’s conformational stability. This comparison demonstrates how silk fibroin’s stability, particularly in silk II, is supported by its β-sheet crystalline network, which is more resistant to disruption than the flexible α-helices in silk I.

#### 3.1.2. Effect of Temperature on C^α^ Atoms Deviations

The influence of temperature on the structural stability of silk fibroin was also evaluated by examining RMSD values over simulated timesteps at temperatures ranging from 298 K to 378 K. Figure 6d depicts the RMSD of the silk I structure, while Figure 6e presents the results for silk II. Figure 6f provides a comparative analysis of average RMSD values for both structures across the range of temperatures simulated. At 298 K, the RMSD values for silk I and silk II were 0.764 Å and 0.742 Å, respectively, indicating relatively stable structures at ambient temperature. However, as the temperature increased to 378 K, the RMSD values rose to 0.871 Å for silk I and 0.843 Å for silk II, reflecting a clear trend of increasing structural deviation with temperature. This suggests that thermal agitation disrupts the stability of the silk fibroin structures, as the higher temperature leads to increased kinetic energy and atom movement within the protein.

Thermal agitation induces vibrational motion of atoms, particularly in the flexible regions of the protein structure, which causes increased backbone deviations. The RMSD results imply that, similar to the effects of pressure, rising temperatures reduce the stability of silk fibroin. However, the magnitude of change is less dramatic compared to the pressure-induced deviations. Silk II again shows slightly more resilience to temperature-induced instability compared to silk I, which may be attributed to its greater β-sheet content. The β-sheets provide a more rigid, crystalline framework that resists thermal fluctuations more effectively than the α-helical and β-turn structures present in silk I.

#### 3.1.3. RMSD Assessment on Structural Dynamics

The results of RMSD analysis suggest that both pressure and temperature variations destabilize the silk fibroin structures to varying degrees, with pressure having a more pronounced destabilizing effect than temperature. Silk II demonstrates more robust structural integrity under both conditions, likely due to its higher β-sheet content, which contributes to enhanced mechanical stability. The observed increase in RMSD values with rising pressure and temperature is indicative of disrupted non-bonded interactions within the silk fibroin structure. At higher pressures, van der Waals interactions and electrostatic forces become less effective at maintaining atomic cohesion, leading to greater deviations from the initial structure. Similarly, thermal agitation at elevated temperatures can disrupt these non-bonded forces, particularly in regions with more flexible secondary structures such as random coils or β-turns.

### 3.2. RMSF Analysis of Silk Structures

**Figure 7:**
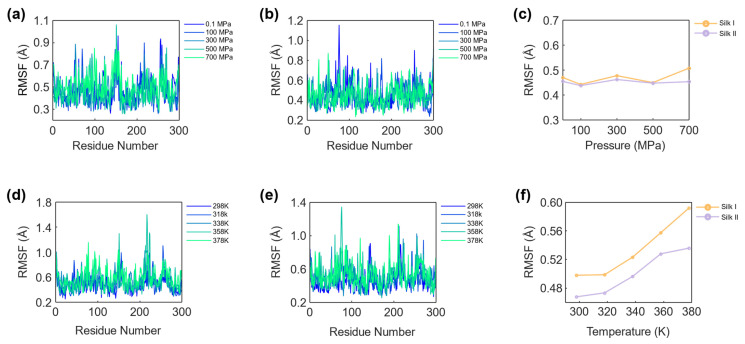
Root Mean Square Fluctuation (RMSF) of silk I and silk II structures under different pressure and temperature conditions. (**a**) RMSF values for silk I backbone atoms across a pressure range of 0.1 MPa to 700 MPa, showing per-residue fluctuations and the effect of pressure on protein flexibility. (**b**) RMSF values for silk II under the same pressure range, highlighting differences in flexibility between the two structures. (**c**) The average RMSF values for silk I and silk II as a function of pressure. The minimum fluctuation for silk I was 0.470 Å at 0.1 MPa, increasing to a maximum of 0.507 Å at 700 MPa. For silk II, the fluctuation values ranged from 0.454 Å to 0.453 Å over the same pressure range. The observed fluctuations are relatively low, indicating minimal atomic mobility under pressure for both structures. (**d**) RMSF values for silk I backbone atoms across a temperature range of 298 K to 378 K, showing increased fluctuations with rising temperature. (**e**) RMSF values for silk II at the same temperature range, reflecting a similar trend of increased fluctuations with temperature. (**f**) The average RMSF values for silk I and silk II as a function of temperature. The fluctuations increase with temperature, with silk I showing a rise from 0.498 Å at 298 K to 0.592 Å at 378 K, and silk II increasing from 0.468 Å to 0.536 Å. These results suggest that temperature-induced thermal agitation leads to increased per-residue flexibility, particularly for silk I, which demonstrates greater fluctuation than silk II.

To further examine the structural stability of silk fibroin under varying external conditions, the Root Mean Square Fluctuation (RMSF) was calculated for both silk I and silk II structures. RMSF provides insight into the flexibility of individual residues by quantifying the extent of atomic displacement relative to their average positions during the simulation. This analysis is crucial for identifying regions within the protein that exhibit higher mobility, which may correlate with structural instability or flexibility. Figure 7 presents the RMSF of the backbone C^α^ atoms in the silk fibroin residues under varying pressure and temperature conditions.

#### 3.2.1. Effect of Pressure on Silk Per-Residue Fluctuations

The impact of pressure on the per-residue fluctuations of the silk I and silk II structures is illustrated in Figure 7a and Figure 7b, respectively. These plots show the RMSF values at pressures ranging from 0.1 MPa to 700 MPa, while Figure 7c provides a comparative evaluation of the average RMSF values for both structures. The RMSF data reveal that neither silk I nor silk II experience significant fluctuations, as reflected by the relatively stable fluctuation in values over the range of simulated pressures. For silk I, the RMSF values range from a minimum of 0.470 Å at 0.1 MPa to a maximum of 0.507 Å at 700 MPa, while silk II shows slightly lower fluctuations, with a minimum value of 0.454 Å at 0.1 MPa and a maximum value of 0.453 Å at 700 MPa. This small difference in fluctuation magnitude suggests that both structures maintain a relatively high degree of stability under pressure, though silk I shows slightly more sensitivity to pressure variations than silk II.

Interestingly, the fluctuation peaks do not follow a uniform trend, with some irregularities observed in the response of different residues to increasing pressure. This variation is likely due to the heterogeneous nature of the protein’s structure, where distinct regions (e.g., β-sheets, α-helices, or loops) react differently to mechanical stress [65]. The β-sheet regions, known for their rigidity, may experience minimal fluctuations, while more flexible regions, such as random coils or turns, exhibit greater movement. The undulating trend in RMSF values across different pressure conditions indicates that structural stability is not uniformly distributed across the protein; instead, it fluctuates on a per-residue basis. This result demonstrates the importance of examining local structural dynamics when assessing the stability of silk fibroin under mechanical stress. Despite the overall stability of the silk fibroin structures, the observed variability in response to pressure across specific residues suggests that the application of pressure can lead to localized structural alterations. These findings are important for applications where silk fibroin may be subjected to mechanical loads or pressures, as they highlight the potential for differential responses in various structural domains of the protein.

#### 3.2.2. Effect of Temperature on Per-Residue Fluctuations

The effect of temperature on the per-residue fluctuations of the silk fibroin structures is shown in Figure 7d for silk I and Figure 7e for silk II, with temperatures ranging from 298 K to 378 K. Figure 7f provides a comparative analysis of the average RMSF values for both silk structures across the range of temperatures simulated. Unlike the relatively subtle response to pressure, both silk I and silk II exhibit more pronounced fluctuations with increasing temperature. For silk I, the RMSF values range from a minimum of 0.498 Å at 298 K to a maximum of 0.592 Å at 378 K, while silk II shows slightly lower fluctuation values, with a minimum of 0.468 Å at 298 K and a maximum of 0.536 Å at 378 K. The overall increase in RMSF values with rising temperature indicates that both silk I and silk II structures undergo greater atomic displacement as thermal agitation intensifies.

This direct relationship between temperature and RMSF can be attributed to the increase in the kinetic energy of the protein atoms as the temperature rises. Higher temperatures provide more energy for atomic vibrations and movements, leading to greater fluctuations in the positions of individual residues. These thermal fluctuations reduce the structural stability of the protein by increasing the likelihood of unfolding or local destabilization, particularly in more flexible regions of the structure. The RMSF plots indicate that silk I exhibits greater fluctuations compared to silk II at all temperatures, suggesting that silk I is more susceptible to temperature-induced instability. This observation is consistent with the known structural characteristics of the two silk types. Silk II, with its higher β-sheet content, forms a more rigid and crystalline structure that is better able to resist thermal agitation. In contrast, silk I contains a higher proportion of flexible α-helices and β-turns, which are more prone to thermal disruption.

Furthermore, the comparative alignment of the average fluctuation signals for both silk structures across different temperatures shows that the residues of the silk protein are highly responsive to temperature variations. As the temperature increases, the individual residues experience greater thermal agitation, leading to a corresponding reduction in stability. This observation highlights the critical impact of temperature on protein dynamics, where elevated temperatures can compromise structural integrity by promoting enhanced flexibility and atomic movement. The results of the RMSF analysis provide valuable insight into the localized dynamics of silk fibroin under different environmental conditions. While both pressure and temperature influence residue fluctuations, the effect of temperature is more pronounced, suggesting that thermal conditions play a more significant role in destabilizing silk fibroin structures. This has important implications for the design and application of silk-based materials, particularly in environments where thermal stability is a key requirement.

#### 3.2.3. RMSF Assessment on Structural Dynamics

The RMSF analysis provides a detailed view of how individual residues within the silk fibroin structures respond to external pressures and temperatures. The fact that both silk I and silk II exhibit minimal fluctuations under pressure suggests that silk fibroin can maintain its stability under mechanical stress, making it suitable for applications involving load-bearing or high-pressure environments. However, the localized fluctuations observed for specific residues indicate that certain regions may be more susceptible to pressure-induced perturbations. On the other hand, temperature variations had a much more substantial impact on the residue fluctuations of both silk structures, particularly for silk I. The increased thermal agitation at higher temperatures leads to a reduction in structural stability, especially in regions containing flexible secondary structures. These findings underscore the importance of temperature control in maintaining the structural integrity of silk fibroin in applications requiring thermal stability. Subsequently, silk II demonstrates better resilience to both pressure and temperature variations, primarily due to its higher β-sheet content, which provides a more stable framework. This suggests that silk II may be better suited for applications where mechanical stability and thermal resistance are critical, while silk I’s greater flexibility may be advantageous in environments where conformational adaptability is desired.

### 3.3. Rg Analysis of Silk Structures

**Figure 8:**
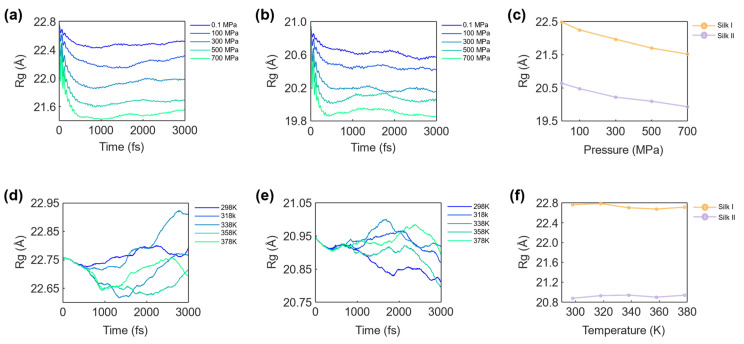
Radius of Gyration (R_g_) of silk I and silk II structures under different pressure and temperature conditions. (**a**) R_g_ values for silk I across a pressure range of 0.1 MPa to 700 MPa, illustrating how pressure affects the overall compactness of the structure. (**b**) R_g_ values for silk II under the same pressure range, showing a similar trend but with lower values compared to silk I. (**c**) The average R_g_ values for silk I and silk II as a function of pressure. An increase in pressure leads to a reduction in R_g_, indicating increased compaction of both structures. Silk I shows a maximum R_g_ of 22.485 Å at 0.1 MPa, decreasing to 21.520 Å at 700 MPa. Silk II exhibits a maximum R_g_ of 20.635 Å at 0.1 MPa, reducing to 19.929 Å at 700 MPa. These results confirm that pressure induces compaction, with silk I showing a greater reduction in compactness than silk II. (**d**) R_g_ values for silk I across a temperature range of 298 K to 378 K, indicating how thermal effects impact protein packing. (**e**) R_g_ values for silk II under the same temperature range, showing minimal changes in compactness. (**f**) The average R_g_ values for silk I and silk II as a function of temperature. Both structures show minimal alterations in compactness with temperature, with R_g_ values of 22.762 Å for silk I and 20.874 Å for silk II at 298 K, slightly decreasing to 22.711 Å and 20.937 Å, respectively, at 378 K. The small changes in R_g_ suggest that the temperature range studied has a negligible effect on the compactness of the silk structures.

Protein stability is closely linked to the spatial arrangement and packing density of its constituent amino acid residues. The Radius of Gyration (R_g_) serves as a metric for determining the overall compactness of a protein, where lower R_g_ values correspond to more tightly packed structures. The tighter packing of amino acids has been shown to enhance both protein stability and folding efficiency, making R_g_ a critical indicator of structural integrity [66]. In this study, we analyzed the R_g_ values for both silk I and silk II structures under varying temperature and pressure conditions to further probe their stability. Figure 8 presents the observed changes in R_g_ for silk I and silk II under different simulation conditions.

#### 3.3.1. Effect of Pressure on R_g_

Figure 8a,b illustrate the R_g_ values of silk I and silk II, respectively, over time at pressures ranging from 0.1 MPa to 700 MPa. The comparative assessment of the average R_g_ values for both silk structures at these pressure levels is depicted in Figure 8c. As pressure increased, a clear decrease in R_g_ was observed for both silk I and silk II. At 0.1 MPa, the maximum R_g_ values for silk I and silk II were 22.485 Å and 20.635 Å, respectively, while at 700 MPa, these values decreased to 21.520 Å for silk I and 19.929 Å for silk II. This reduction in R_g_ suggests that both silk fibroin structures experienced increased compaction as pressure increased, consistent with the compression of interatomic distances.

The more substantial reduction in R_g_ for silk I compared to silk II can be attributed to the inherent structural differences between the two. Silk II, with its higher content of β-sheets, has a more tightly packed, crystalline structure, while silk I, dominated by α-helices, is relatively more flexible and less compact under low-pressure conditions. As pressure increases, the more flexible silk I structure undergoes greater structural compaction due to the rearrangement and tighter packing of its secondary structural elements. In contrast, the more rigid silk II structure is already highly compact due to its predominant β-sheet content, resulting in a less-pronounced reduction in R_g_ under pressure. These results indicate that pressure plays a significant role in determining the overall compactness of silk fibroin structures, with silk I being more sensitive to pressure-induced compaction than silk II. This finding is particularly relevant for applications where silk fibroin is subjected to high-pressure environments, as the structural integrity and compactness of the protein may be directly influenced by the applied mechanical stress.

#### 3.3.2. Effect of Temperature on R_g_

Figure 8d,e display the R_g_ values of silk I and silk II, respectively, under varying temperatures from 298 K to 378 K, while Figure 8f provides a comparative analysis of the average R_g_ values for both structures at these temperature levels. In contrast to the significant changes observed under pressure, the variation in R_g_ with temperature was minimal for both silk I and silk II. At 298 K, the R_g_ values were 22.762 Å for silk I and 20.874 Å for silk II, and at 378 K, the values were 22.711 Å for silk I and 20.937 Å for silk II. The minor changes in R_g_ indicate that the temperature range employed in this study (298 K to 378 K) had little impact on the compactness of either silk structure.

The relatively small variation in R_g_ with temperature can be explained by the insufficient thermal agitation within this range to significantly alter the protein’s compactness. Protein unfolding or expansion typically occurs at higher temperatures, often exceeding 500 K, where sufficient thermal energy disrupts non-bonded interactions, leading to structural expansion or unfolding [67,68]. In the present study, the simulated temperature range engulfs the typical processing conditions for silk fibroin in various engineering applications [34], where maintaining structural integrity is essential. Thus, it is important to note that the thermal conditions simulated here reflect a realistic temperature range for practical applications, such as biomedical devices and tissue engineering scaffolds, where silk fibroin must retain its structural compactness and stability. Moreover, the modest increase in kinetic energy within this temperature range was insufficient to induce substantial changes in the packing of the silk fibroin structures. Hence, the absence of significant changes in Rg suggests that both silk I and silk II maintain their structural integrity and compactness under the temperatures typically encountered in fabrication processes.

#### 3.3.3. R_g_ Assessment on Structural Dynamics

The R_g_ analysis provides valuable insights into the structural behavior of silk fibroin under different environmental conditions. The significant reduction in R_g_ with increasing pressure highlights the sensitivity of silk fibroin to mechanical compression, with silk I showing a greater degree of compaction due to its more flexible α-helix-dominated structure. In contrast, silk II, which is more rigid due to its β-sheet content, exhibited a more modest reduction in R_g_, reflecting its inherently compact nature. Conversely, temperature variations had a minimal effect on the compactness of both silk I and silk II within the simulated range, indicating that the silk fibroin structures are thermally stable under these conditions. This finding is consistent with the relatively low temperatures simulated, which were insufficient to induce significant thermal expansion or unfolding. The results suggest that silk fibroin is well-suited for applications where temperature stability is crucial, such as in biomedical devices or high-performance materials that must maintain their structural integrity across a range of temperatures. Furthermore, the R_g_ analysis confirms that silk fibroin’s structural compactness is highly sensitive to pressure but remains stable under moderate temperature variations. Silk II, with its greater β-sheet content, exhibits enhanced stability under both conditions, making it a more suitable candidate for applications requiring mechanical and thermal stability. Silk I, while more flexible, may be advantageous in applications where adaptability and structural flexibility are desired.

### 3.4. Comparative Assessment of Silk Structural Dynamics

The temperature and pressure ranges selected for this study were chosen to closely reflect the practical and experimental conditions relevant to silk fibroin processing and structural stability investigations. The temperature range of 298 K to 378 K corresponds to typical conditions used in the degumming process of silk fibroin extraction. Degumming is a critical step in silk processing, wherein silk cocoons are boiled in a sodium carbonate solution to remove sericin [34], a gummy protein that binds silk fibroin filaments. Temperatures in this process typically span from ambient room temperature (298 K or 25 °C) up to 378 K (105 °C), the upper limit frequently applied during boiling. By simulating silk fibroin structures within this range, we aimed to provide insights into the thermal stability of silk fibroin as it might be influenced by processing temperatures, offering a direct connection between simulation conditions and real-world applications.

The pressure range, extending to 700 MPa, was selected based on prior studies that utilized high-pressure experimental techniques in combination with molecular dynamics simulations to explore protein structural responses to mechanical stress. Pressures in this range have been shown to induce conformational changes without causing denaturation, which provides a realistic model for examining protein stability under stress [33,61,69]. For silk fibroin, understanding its stability under such conditions is particularly relevant due to its potential applications in environments where mechanical resilience is essential, such as in load-bearing biomedical scaffolds or high-pressure processing environments. These conditions allow us to probe subtle structural rearrangements in silk fibroin, such as the effect on non-bonded interactions, hydrogen bonding, and compactness, which are critical for its application in stress-bearing or dynamic environments. By choosing a pressure range that is both scientifically validated and practically relevant, our study provides insights that can bridge theoretical analysis with real-world applications.

The primary factors influencing protein packing and stability are the amino acid composition and the secondary structure [66]. In the case of silk fibroin, while both silk I and silk II structures share the same amino acid composition, their secondary structural configurations differ significantly, driving the observed variations in their responses to external conditions such as temperature and pressure. The statistical analyses of protein structures indicate that α-helix-dominated proteins typically exhibit a larger Radius of Gyration (R_g_) and faster folding rates compared to β-sheet and α/β-protein structures [66]. This suggests that α-helix structures are generally less tightly packed, making them more prone to structural instability and conformational changes when subjected to external stimuli. The results of this study clearly demonstrate these differences in stability between silk I and silk II. The silk I structure, dominated by α-helices and β-turns, exhibits greater sensitivity to environmental changes. This is evidenced by the larger deviations observed in the Root Mean Square Deviation (RMSD) and greater fluctuations in the Root Mean Square Fluctuation (RMSF) plots across varying temperature and pressure conditions. The α-helical domains in silk I are inherently more flexible and less rigid than the β-sheets that dominate the silk II structure. This flexibility translates into increased atomic displacements and greater conformational fluctuations when the silk I structure is exposed to stressors such as elevated pressure or temperature.

In contrast, the silk II structure, which is characterized by a higher β-sheet content, demonstrated significantly greater stability under the same conditions. The tightly packed, crystalline nature of β-sheets provides silk II with superior structural integrity, making it more resistant to both mechanical and thermal perturbations. The β-sheets form a highly ordered and stable network of hydrogen bonds, which restricts atomic movement and maintains structural cohesion even under elevated pressure or temperature. This is reflected in the lower RMSD and RMSF values for silk II compared to silk I, indicating that the former undergoes fewer deviations and fluctuations under the same conditions. The R_g_ analysis further supports this comparative assessment. Silk I exhibited larger R_g_ values, indicating a less compact structure that is more prone to expansion and structural rearrangement under stress. The pressure-induced reduction in R_g_ was more pronounced in silk I, suggesting that it experiences greater compaction under mechanical stress, a result of its initial looser packing. On the other hand, silk II, with its more compact β-sheet-dominated structure, exhibited smaller R_g_ values and less variation in response to pressure changes, indicating that it remains more stable and resists compression.

These results demonstrate the critical role of the secondary structure in determining the stability of silk fibroin. Regardless of the external conditions applied—whether varying temperature or pressure—silk I consistently demonstrated larger deviations and fluctuations compared to silk II, signifying its reduced stability. The increased sensitivity of silk I to environmental changes makes it less suited for applications that require high mechanical and thermal stability. In contrast, silk II’s superior stability under pressure and temperature variations makes it a more robust candidate for applications where structural integrity is paramount, such as in biomedical scaffolds, drug delivery systems, or other high-performance materials that are exposed to dynamic environmental conditions. Therefore, the difference in the secondary structure between silk I and silk II is the primary driver of their differing responses to external conditions. The α-helical structure of silk I contributes to its greater flexibility and lower stability, while the β-sheet structure of silk II confers enhanced stability and compactness, making it more resilient to external perturbations.

Previous molecular dynamics studies have extensively demonstrated how temperature and pressure affect protein structure and stability through distinct mechanisms. Elevated temperatures tend to enhance protein flexibility but often at the expense of structural stability, as seen in proteins such as dihydrolipoamide dehydrogenase [70] and orthologous proteins [32]. In these studies, MD simulations have shown that heat induces greater molecular motion, leading to a loosening of compact structures or partial unfolding. By loosening internal molecular packing, temperature-driven flexibility can compromise structural cohesion, particularly in proteins with a significant helical content, similar to the silk I structure in our study. Furthermore, our findings align with studies of thermally adapted proteins, such as those from cold-adapted organisms, where stability is preserved by optimizing structural flexibility for lower temperatures. This adjustment highlights a critical trend in protein stability: proteins tend to trade structural rigidity for flexibility under thermal stress, leading to different stability outcomes depending on secondary structure composition. In the case of silk I, this trend translates to greater susceptibility to destabilization under temperature variations, consistent with previously reported MD observations on helical versus β-sheet-dominated structures.

High pressure, as demonstrated in prior MD studies, typically induces localized structural changes in proteins, often without causing full denaturation. These pressure-induced perturbations are frequently observed near functional sites and flexible regions, where they can alter structural dynamics without entirely disrupting protein function. For example, pressure studies on green fluorescent protein mutants have revealed conformational changes affecting fluorescence properties, illustrating how pressure can selectively impact structural regions without extensive unfolding [33]. Studies on small proteins such as chignolin further illustrate that temperature and pressure impact protein stability through different pathways—temperature-driven unfolding primarily increases entropy, while pressure-induced denaturation results from water penetrating hydrophobic regions [69]. In line with these trends, our findings show that silk fibroin’s β-sheet-rich silk II structure is more resistant to pressure-induced perturbations compared to the more flexible α-helical structure of silk I. This is consistent with established findings that compact, β-sheet structures provide greater resilience to pressure due to their tighter packing and extensive hydrogen bonding. Our results reinforce the importance of the secondary structure in defining protein stability, highlighting how pressure and temperature modulate protein behavior in complementary yet distinct ways.

Both temperature and pressure are critical factors that influence the stability and performance of silk fibroin, with their relative importance varying according to the intended application or processing technique. In applications that involve thermal processing, such as thermal modification of silk-based materials, temperature stability is paramount. Silk fibroin structures must be able to maintain their integrity and functionality within the relevant temperature range to ensure effective application. Silk II, with its β-sheet-dominated structure, exhibits higher thermal stability than silk I, making it a more suitable candidate for high-temperature applications that demand resilience against thermal fluctuations. Conversely, in high-pressure applications, such as those involving mechanical reinforcement in load-bearing devices or high-pressure processing, pressure resilience is of primary importance. Silk fibroin’s behavior under pressure is crucial for applications where mechanical stress is prevalent, such as in biomedical scaffolds that endure substantial mechanical loads. The structural integrity of silk II, with its compact β-sheet arrangement, may offer advantages under high-pressure conditions, whereas the α-helical structure of silk I, though more flexible, may exhibit greater deformation under similar pressures. The distinct structural characteristics of silk I and silk II thus provide a degree of flexibility in selecting the optimal conformation based on application-specific requirements. This adaptability in structural properties of silk fibroin emphasizes the value of molecular simulations in understanding its stability across different environmental conditions, guiding the development of silk-based materials tailored to diverse biomedical and engineering applications.

### 3.5. Non-Bonded Interactions of Silk Structures

Non-bonded interactions, particularly van der Waals (VDW) forces and electrostatic potential energy (EE), play a crucial role in determining the intermolecular dynamics and stability of protein structures. These interactions, being long-range and non-covalent, are sensitive to changes in temperature and pressure, impacting the behavior of charged atoms and molecules within the protein. To further understand the stability and structural behavior of silk fibroin (silk I and silk II), we analyzed the energy evolution of VDW and electrostatic potential energy under varying pressure and temperature conditions. The pressure simulations were performed under the NPT ensemble from 0.1 MPa to 700 MPa, at a constant temperature of 298 K, while temperature variations were conducted under the NPT ensemble from 298 K to 378 K, at a constant pressure of 1 atm. After optimization of the simulated systems, we analyzed the non-bonded interactions to assess how external conditions affect silk fibroin stability. The surrounding chemical environment plays a crucial role in influencing protein interactions. By conducting the simulations in a uniform aqueous environment, we effectively isolated the effects of temperature and pressure, ensuring that any observed energy variations were solely due to these parameters. This approach enabled a focused examination of van der Waals and electrostatic interactions in silk fibroin stability, free from interference by additional chemical factors, and provided a clearer understanding of how these forces respond to environmental changes.

#### 3.5.1. Effect of Pressure on Non-Bonded Interactions

**Figure 9:**
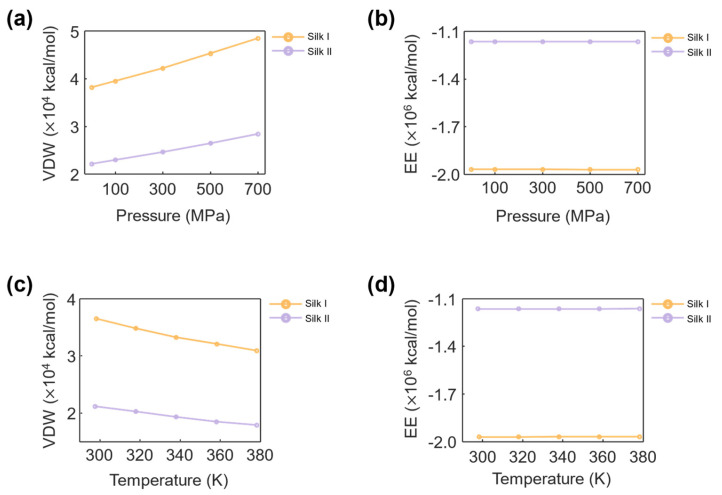
Non-bonded interactions of silk I and silk II structures under different pressure and temperature conditions. (**a**) The average van der Waals energy as a function of pressure, from 0.1 MPa to 700 MPa. For both silk I and silk II, the van der Waals energy increased with rising pressure, reflecting the compression of inter-atomic distances and the strengthening of long-range non-bonded interactions. (**b**) The average electrostatic potential energy as a function of pressure. The electrostatic potential energy remained constant for both silk I and silk II, with values of −1.97 × 10^6^ kcal/mol and −1.16 × 10^6^ kcal/mol, respectively, indicating that pressure has no significant effect on electrostatic interactions. (**c**) The average van der Waals energy as a function of temperature, from 298 K to 378 K. In contrast to pressure, the van der Waals energy decreased with increasing temperature for both silk structures, due to the expansion of inter-atomic distances and the weakening of non-bonded interactions. (**d**) The average electrostatic potential energy as a function of temperature. Similar to pressure, the electrostatic potential energy remained constant with temperature for both silk I and silk II, highlighting the stability of long-range electrostatic interactions under thermal fluctuations.

The effect of pressure on the non-bonded interactions of silk I and silk II structures is shown in Figure 9. As the pressure increased, the van der Waals energy increased for both structures. Figure 9a illustrates that for silk I, the van der Waals energy rose from 3.82 × 10^4^ kcal/mol at 0.1 MPa to 4.85 × 10^4^ kcal/mol at 700 MPa. Similarly, for silk II, the energy increased from 2.22 × 10^4^ kcal/mol to 2.84 × 10^4^ kcal/mol over the same pressure range. This increase in van der Waals energy can be attributed to the compression of the inter-atomic distances as pressure rises, which in turn intensifies the weak, long-range interactions between atoms. As the pressure compresses the protein structure, the atoms are brought closer together, amplifying the van der Waals forces and increasing the overall energy associated with these interactions.

The observed trend is consistent with the nature of van der Waals interactions, which are highly sensitive to the distances between atoms. These forces, although relatively weak compared to covalent bonds, become significant when atoms are brought into close proximity, as occurs under high pressure. The increase in van der Waals energy under pressure indicates that both silk I and silk II structures undergo compaction, which reinforces their internal interactions. However, silk I exhibits higher van der Waals energy than silk II, reflecting the greater flexibility and atomic mobility of the silk I structure, which is dominated by α-helices rather than the more rigid β-sheet structure of silk II.

In contrast, the electrostatic potential energy remained constant across the entire range of pressure variations for both silk I and silk II. As shown in Figure 9b, the electrostatic potential energy for silk I was approximately −1.97 × 10^6^ kcal/mol, while for silk II, it was −1.16 × 10^6^ kcal/mol, regardless of pressure. Electrostatic interactions are primarily governed by the distribution of charges within the protein and between the protein and its environment. In this study, the proteins were simulated in a neutral aqueous environment, with no external counterions or charged species that could significantly alter the charge distribution. As a result, the electrostatic potential energy, which is derived from conservative Coulombic forces, remained unaffected by the changes in pressure.

The stability of electrostatic potential energy suggests that the charged residues in silk fibroin are not significantly influenced by mechanical compression. The distribution and proportion of charged and polar residues on the silk fibroin surface determine its electrostatic properties [71]. Since the protein was immersed in water and no chemical reactions or ionization occurred, the electrostatic behavior remained stable, independent of pressure changes. This result indicates that the long-range electrostatic interactions within silk fibroin are resilient to mechanical perturbations, providing a stable electrostatic environment even under high-pressure conditions.

#### 3.5.2. Effect of Temperature on Non-Bonded Interactions

The effect of temperature on the non-bonded interactions of silk fibroin is presented in Figure 9. Unlike pressure, an inverse relationship was observed between the temperature and van der Waals energy. Figure 9c shows that for silk I, the van der Waals energy decreased from 3.65 × 10^4^ kcal/mol at 298 K to 3.09 × 10^4^ kcal/mol at 378 K, while for silk II, the energy dropped from 2.12 × 10^4^ kcal/mol to 1.79 × 10^4^ kcal/mol over the same temperature range. This decline in the van der Waals energy with increasing temperature can be attributed to the thermal agitation of atoms within the protein structure. As the temperature rises, the atomic motion increases, which leads to the expansion of inter-atomic distances and a reduction in the strength of van der Waals interactions. The increased kinetic energy causes the atoms to vibrate more, reducing the close packing needed for the van der Waals forces to be effective. Consequently, the overall van der Waals energy decreases as the temperature rises, indicating that thermal agitation weakens the compaction of the silk fibroin structures.

The effect of temperature on electrostatic potential energy, as observed in Figure 9d, was negligible. For both silk I and silk II, the electrostatic potential energy remained unchanged, with silk I at −1.97 × 10^6^ kcal/mol and silk II at −1.16 × 10^6^ kcal/mol across the temperature range of 298 K to 378 K. Similar to the observations for pressure, the electrostatic interactions were unaffected by temperature variations. Since the electrostatic properties are largely determined by the fixed charge distribution of the protein’s residues and the neutral simulation environment, the long-range interactions governed by electrostatic forces remained constant despite thermal fluctuations. The consistency of electrostatic potential energy under varying temperatures suggests that the silk fibroin’s charge distribution remains stable within the temperature range commonly used in processing and fabrication. Proteins generally require extreme temperatures, beyond the range studied here, to significantly alter electrostatic behavior through ionization or charge redistribution [64,65]. Thus, the silk fibroin structures maintain stable electrostatic interactions even in the presence of moderate thermal agitation.

#### 3.5.3. Comparative Assessment of Non-Bonded Interactions

When comparing the non-bonded interactions of silk I and silk II, several key distinctions arise. First, silk I consistently exhibited higher van der Waals energy compared to silk II under both pressure and temperature conditions. This difference can be explained by the structural differences between the two forms. Silk I, with its α-helix-dominated structure, is more flexible and less tightly packed, allowing for greater inter-atomic interactions that amplify the van der Waals forces. In contrast, silk II’s β-sheet-dominated structure is more rigid and compact, reducing the effective range of van der Waals interactions. Conversely, silk II exhibited higher electrostatic potential energy than silk I. This difference suggests that the charged and polar residues in silk II are more distributed or exposed, contributing to stronger Coulombic interactions. The conformation of silk II, which features a more crystalline arrangement of β-sheets, may promote a more favorable charge distribution that increases electrostatic potential. These variations in non-bonded interactions highlight how the structural conformation of silk fibroin significantly influences its molecular interactions. Subsequently, the non-bonded interactions within silk fibroin are heavily influenced by external conditions, with the van der Waals forces being highly sensitive to changes in pressure and temperature, while the electrostatic interactions remain stable. Silk I, being more flexible and less compact, shows higher van der Waals energy, whereas silk II’s rigid β-sheet structure leads to more pronounced electrostatic interactions. Notably, the observed linear trend is consistent with existing studies on protein molecular dynamics that have conducted energy analyses, including van der Waals and electrostatic interactions [70]. These findings are essential for understanding the molecular stability of silk fibroin under various environmental conditions, particularly for applications where mechanical or thermal stability is critical.

## 4. Conclusions

In this study, we conducted Molecular Dynamics (MD) simulations to investigate the structural stability of silk fibroin (silk I and silk II) under varying temperature and pressure conditions. By examining key parameters including RMSD, RMSF, and R_g_, we assessed the response of the silk fibroin structures to external perturbations. Our findings reveal that both temperature and pressure significantly impact the stability of silk I and silk II, though silk I is generally less stable than silk II due to the differences in their secondary structures. Silk I, characterized by a predominance of flexible α-helices, exhibited greater deviation and fluctuation in response to increasing temperature and pressure.

Specifically, RMSD values for silk I increased from 0.804 Å at 0.1 MPa to 1.454 Å at 700 MPa, and from 0.764 Å at 298 K to 0.871 Å at 378 K, indicating its higher susceptibility to environmental changes. In contrast, silk II, dominated by the more rigid β-sheet structures, demonstrated better resilience, with RMSD values rising from 0.772 Å at 0.1 MPa to 1.285 Å at 700 MPa, and from 0.742 Å at 298 K to 0.843 Å at 378 K. The reduced flexibility of silk II, as indicated by lower RMSF values and a more compact structure (with R_g_ decreasing from 20.635 Å at 0.1 MPa to 19.929 Å at 700 MPa), reinforces its superior stability compared to silk I. Non-bonded interactions responded distinctively to environmental changes: the van der Waals energy increased with pressure but decreased with temperature, reflecting the compacting and expansion effects on silk fibroin. Meanwhile, the electrostatic potential energy remained constant, indicating that long-range electrostatic interactions are minimally affected by these external conditions.

The difference in stability between silk I and silk II can be largely attributed to their secondary structures. Silk II, with its dense network of hydrogen-bonded β-sheets, is inherently more resistant to deformation, while silk I’s α-helices confer greater flexibility but reduced stability under stress. This structural distinction has important implications for the design of silk fibroin-based materials, as silk II’s superior stability may make it more suitable for applications requiring high mechanical or thermal stability, whereas silk I’s flexibility could be advantageous in environments where adaptability is needed. The insights provided by this study advance our theoretical understanding of silk fibroin at the atomic level, demonstrating how external conditions such as temperature and pressure influence its structural behavior. Subsequently, models that accurately represent the chemical composition and structural characteristics of silk fibroin are likely to produce results consistent with our findings. This research also demonstrates the utility of computational techniques in predicting the molecular dynamics of protein structures, which can inform experimental approaches and material design. Future work could extend these findings by investigating silk fibroin’s interaction with ions or other molecules to enhance our understanding of its behavior in biological environments.

## Data Availability

The original contributions presented in the study are included in the article, further inquiries can be directed to the corresponding authors.
